# Processing of Al_2_O_3_-AlN Ceramics and Their Structural, Mechanical, and Tribological Characterization

**DOI:** 10.3390/ma14206055

**Published:** 2021-10-14

**Authors:** Dheeraj Varanasi, Monika Furkó, Katalin Balázsi, Csaba Balázsi

**Affiliations:** Institute of Technical Physics and Materials Science, Centre for Energy Research, Eötvös Loránd Research Network, Konkoly-Thege Miklos Utca 29-33, 1121 Budapest, Hungary; dheeraj.varanasi@ek-cer.hu (D.V.); furko.monika@ek-cer.hu (M.F.); balazsi.katalin@ek-cer.hu (K.B.)

**Keywords:** aluminum oxy-nitride, aluminum nitride, hot isostatic pressing, ceramics, tribology

## Abstract

The aim of this study is to present a novel, lower sintering temperature preparation, processing, structural, mechanical, and tribological testing of the AlN-Al_2_O_3_ ceramics. The precursor powder of AlN was subjected to oxidation in ambient environment at 900 °C for 3, 10, and 20 h, respectively. These oxidized powders were characterized by SEM and XRD to reveal their morphology, phase, and crystal structure. The SEM results showed coarse powder particles and the presence of aluminum oxide (Al_2_O_3_) phase at the surface of aluminum nitride (AlN). The XRD analysis has shown increasing aluminum-oxy-nitride conversion of aluminum nitride as the holding time of oxidation increased. The highest percentage of conversion of AlN powder to AlN-Al_2_O_3_ was observed after 10 h. Simultaneously the powders were compacted and sintered using the hot isostatic pressing (HIP) under inert environment (N_2_ gas) at 1700 °C, 20 MPa for 5 h. This led to the compaction and increase in density of the final samples. Mechanical tests, such as bending test and tribology tests, were carried out on the samples. The mechanical properties of the samples were observed to improve in the oxidized samples compared to the precursor AlN. Moreover, applying longer oxidation time, the mechanical properties of the sintered samples enhanced significantly. Optimum qualitative (microstructure, oxide percentage) and quantitative (tribology, hardness, and bending tests) properties were observed in samples with 10-h oxidation time.

## 1. Introduction

Aluminum oxynitrides (AlON) are relatively hybrid ceramic structures of aluminum oxide and aluminum nitride (Al_2_O_3_-AlN). They were first observed in 1959, when Yamaguchi and Yanagida identified the existence of aluminum ion in a spinel like structure under presence of nitrogen/nitride phase forming aluminum oxynitride. To obtain the structure, they concluded the experiments need to be carried out at 1650 °C or higher under reducing environments/vacuum [1]. Similar studies were performed in the next two decades [2,3,4]. In the 1970s–1980s, commercial interest in this material grew and the commercialization was taken up by the scientists of USA, France, and Japan. These researchers observed formation of stable spinel-like phases in oxyntrides. The first known equilibrium phase diagram for AlON spinel phase formed between Al_2_O_3_-AlN system was developed and a stable equilibrium phase configuration was provided for spinel AlON, Al_23_O_27_N_5_ [5,6,7].

This stable phase has a cubic spinel structure called γ-AlON and its properties were widely discussed [8,9,10]. They even gave the compositional limit for the ratio of Al_2_O_3_ and AlN for a successful fabrication of AlON as 64.3 mol% to 35.7 mol%, respectively [10]. AlON is a unique material whose range of properties are yet to be identified and studied. The current research on the oxynitride ceramics is mostly focused on optical properties of the material; however, this needs to be extended to other commercial applications as well. The wide range of applications of AlON extends to armor, EM domes and windows, aircraft and missile domes, IR windows, and semi-conductor processing applications besides others [5]. AlON has a unique crystal structure which enables the material to be flexible in use for various applications as mentioned above. It can be understood as a substitution of nitrogen for oxygen in Al_2_O_3_ or vice-versa. This substitution stabilizes the new phases differing in crystal symmetry and structure leading to α-Al_2_O_3_, AlON, and AlN exhibiting rhombohedral, cubic, and hexagonal structure respectively. AlON is generally also termed as the cubic aluminum oxide stabilized by nitrogen resulting in a cubic spinel structure. This helps in obtaining a transparent ceramic with proper processing technique [10]. The first patent in successfully developing a stable AlON phase material for commercial viability was filed by Raytheon group [11,12] and their optical and mechanical properties were further investigated by McCauley [12,13].

The fabrication of aluminum-based oxy nitrides needs investigation, with increased focus and attention being given to the material due to its potential applications. The main focus needs to be kept on obtaining cost-effective, dense, and compact AlN-Al_2_O_3_ composite ceramics. All the currently known techniques yield oxy-nitride grain size of 150–200 μm. In the future, the interest will be on obtaining nano-sized grains with improved properties and performance [8,9]. The comprehensive overview of various manufacturing techniques was taken up in the recent years [14,15]. Earlier techniques for fabrication of AlON ceramics included reaction sintering [16,17,18], high temperature reactive synthesis, which is initiated by a combustion reaction between the precursors [19,20], and carbothermal synthesis [21,22,23,24,25,26]. Merzhanov [19] and Borovinskaya et al. [20] have investigated with self-propagation of Al-Al_2_O_3_ combustion reaction under nitrogen at high pressure (10–100 MPa) for synthesis of AlN-Al_2_O_3_ composite ceramic. In the carbothermal synthesis performed by Zheng et al. [21], the precursor aluminum oxide and carbon powders were heated in a crucible under nitrogen gas at temperatures between 1600 °C and 1900 °C. The carbon escapes in form of gas upon reacting with excess oxygen and nitrogen flowing through, forming AlON phase [21]. More recent studies include synthesis of precursor powders by sintering method and later using carbothermal synthesis to obtain the target composite AlN-Al_2_O_3_ ceramic substrates [22,23,24,25,26].

Among other methods, pressureless sintering and spark plasma sintering were also tested resulting in formation of high quality Al-O-N phase ceramics [27,28,29,30,31,32,33,34]. Zhang et al. [27] prepared the AlON ceramic using single step pressureless sintering. The precursors used were α-Al_2_O_3_ and aluminum powders. The aluminum powders were nitridized into highly active AlN under the flowing nitrogen. They concurrently reduced the sintering temperature and exhibited that the cheaper Al_2_O_3_ and aluminum powders can be acceptable substitutes. The optimum sintering process was limited at 1750 °C for 2 h. Higher temperatures could not be used here because of the subsequent displacement of oxygen by nitrogen atoms at higher temperatures. Shan et al. [28,29] have developed Al-oxynitride ceramics by pressureless sintering method using ball milled AlN-Al_2_O_3_ powder as precursor. They have reported obtaining denser sintered ceramic substrates when processed at 1880 °C for time between 90 and 150 min. Similarly, AlON ceramics were produced using Spark Plasma Sintering (SPS). Particularly Shan et al. [31] used infrared SPS using γ-AlON powders produced from carbothermal synthesis at temperatures between 1350 and 1500 °C at pressure of 40 MPa. The samples were densified within 10 min with 78% optical transparency. Sahin et al. [33] used spark plasma sintering using mixture of Al_2_O_3_ and AlN powder mixtures processed at temperatures between 1400–1650 °C at 40 MPa under N_2_ environment. They demonstrated that a sintering process above 1650 °C for a duration longer than 2 h will most certainly result in dense, pure AlON ceramics.

Alternative to sintering, more novel approaches to obtain denser γ-AlON phase ceramics are by pressing—hot pressing and isostatic pressing. The difference in both the methods is in the direction and magnitude of application of pressure alongside the simultaneous application of heat. The advantage of hot isostatic pressing (HIP) over conventional sintering processes is in obtaining very high-quality end products. Comparatively, HIP requires lower temperatures and shorter times for obtaining denser Al-O-N ceramic products. This makes HIP an interesting and exciting research processing technique [15]. The HIP usually is followed by sintering of the powders and includes additives called sintering additives which provide the required thermal stability and help in achieving densification during the processing [35,36,37,38]. The most common additives used during HIP are La_2_O_3_/Y_2_O_3_ [36,37] while an alternative SiO_2_ additive was also researched for being a sintering additive [38]. Chen et al. [36,37] stated that sintering additives have a huge role in the final porosity of the sintered AlON substrates. They found that instead of using individual Y_2_O_3_ or La_2_O_3_ additives, co-doping of both provides more effective and efficient sintered AlON with lower porosity. These substrates then can easily be densified during the HIP processing. The amount of additives required to achieve full densification were 0.08 w% Y_2_O_3_ and 0.02 w% La_2_O_3_. Hot isostatic pressing is usually carried out at higher temperatures in the range of 1600 °C to 1900 °C under an inert gas environment like Ar under high pressures usually around 150–200 MPa [39].

The current paper is focused on the novel eco-friendly, comparatively low temperature fabrication of AlN-Al_2_O_3_ composite ceramics using the two-step oxidation and HIP technology. The aim of the investigations is to produce the AlN-Al_2_O_3_ composite in an environmentally friendly method. The current method has the advantages of lower processing temperature/time and energy requirements. The qualitative analysis of the obtained ceramic phase was analyzed by electron microscopy (SEM) and X-ray diffraction (XRD) techniques to observe the phase, composition, and microstructure of the final AlN-Al_2_O_3_ composite. The samples were also tested for their mechanical and tribological properties.

## 2. Materials and Experimental Methods

### 2.1. Materials

AlN powder (H.C. Starck GMBH, Berlin, Germany) with 99% purity was used as precursor material. The particle size of the precursor powder was 1.3 ± 0.5 µm and the specific surface area was 6 ± 2 μm^2^/g.

### 2.2. Experimental Methods

#### 2.2.1. Oxidation and Sintering of Powders

The powders were oxidized in ambient atmosphere at 900 °C for 3, 10, and 20 h respectively. The oxidized powders were subjected to simultaneous sintering using Hot Isostatic Pressing (HIP, ABRA type) at pressure of 20 MPa, 1700 °C under an inert gas (nitrogen) as static environment for 5 h as a holding time with a heating rate of 25 °C/min in graphite tank.

#### 2.2.2. Density Measurements

The apparent density of the composites was measured using Archimedes’ method (Equation (1)) with water as the immersion medium. Some composites with high surface porosity have been immersed into water + lubricant (soap) for at least 3 days to ensure the total filling of the pores.

(1) $$\mathrm{Apparent}\;\mathrm{density}=\frac{\mathrm{wt}.\;\mathrm{of}\;\mathrm{dry}\;\mathrm{sample}}{\mathrm{wt}.\;\mathrm{of}\;\mathrm{dry}\;\mathrm{sample}-\mathrm{wt}.\;\mathrm{of}\;\mathrm{soaked}\;\mathrm{immersed}\;\mathrm{sample}}.\rho_{\mathrm{Water}}$$

where $\rho_{\mathrm{Water}}$ is water density equal to 1 g/cm^3^ at room temperature and (wt.) refers to the weight in (g). Five samples from each series were measured and their average value was indicated with error ±2%.

#### 2.2.3. Mechanical Tests

The HIP sintered samples were subjected to mechanical tests, such as bending tests (3-point and 4-point), hardness, and tribology tests to quantify the properties. Bending tests were carried out on a machine equipped with both 3-point and 4-point bending setup and a load of 10 KN. In this work, 3-point bending test was conducted using apparatus (INSTRON5966, Darmstadt, Germany) with a span of 20 mm and rate of 0.0083 mm/s with sample size 5 mm × 5 mm × 30 mm for 3 samples/type and 5 mm × 5 mm × 50 mm for 3 samples/type for 4-point bending test, as well. The samples were prepared for the bending tests in the required dimensions and subjected to loading to carry out the tests.

Hardness test was carried out with sintered samples on Leitz Wetzlar 721,464 Vickers micro hardness testing equipment (Wetzlar, Germany) using load of 19.61 N (2000 P). The hardness was calculated from the diagonal length indentation according to the equation below, Equation (2).

(2) $$H_{v}=\frac{1.89\cdot F\cdot 10^{3}}{d^{2}}$$

*H_v_* Vickers hardness, *F* is force applied (N), and d is the diagonal length (mm).

#### 2.2.4. Tribological Characterization

Tribology tests were conducted on the sintered substrates to observe the wear rate (V, mm^3^/Nm) under dry sliding conditions using ball on disc experimental mechanism. The discs were sintered AlN-Al_2_O_3_ samples and silicon nitride (Si_3_N_4_) ball of 5 mm diameter was used as a sliding counter body. A total sliding distance of 2000 m was performed with a static load of 5 N, 500 rpm at room temperature.

The volumetric wear can be calculated from track width (after the experiment) using the following equation, Equation (3).

(3) $$V_{disc}=2\pi R\left[r^{2}\sin^{-1}\left(\frac{d}{2r}\right)-\left(\frac{d}{4}\right)\sqrt{\left(4r^{2}-d^{2}\right)}\right]$$

where *r* is the ball radius (mm), *d* is the width of the track on disc (mm), *R* is the radius of the track on the disc (mm), and *V_disc_* is the volume wear of the disc (mm^3^).

Upon calculating the volumetric wear, Wear Factor can be calculated from Equation (4).

(4) $$K=\frac{V_{disc}}{Fl}$$

*F* is force (N), *l* is the sliding distance (m), and *K* has units of mm^3^/Nm.

#### 2.2.5. Microstructure and Morphological Characterization

The oxidized powders, sintered substrates, and tribological wear tracks on the sintered substrates were analyzed for their microstructural morphology.

Optical microscopy has been carried out by Keyence digital-optical microscope, (Keyence VHX-950F model, Osaka, Japan). Scanning Electron Microscopy has been carried out by using Thermo Scientific Scios 2 equipment (Waltham, MA, USA) under SEM-BSD and SEM-SE modes using 4kV accelerating voltage. Phase analysis and elemental identification was performed by XRD—Cu Kα radiation using a Bruker AXS D8 Discover diffractometer (Billerica, MA, USA) equipped with Göbel-mirror and a scintillation detector.

## 3. Results and Discussion

### 3.1. Oxidation of AlN Powders and Subsequent HIP Sintering

**Figure 1:**
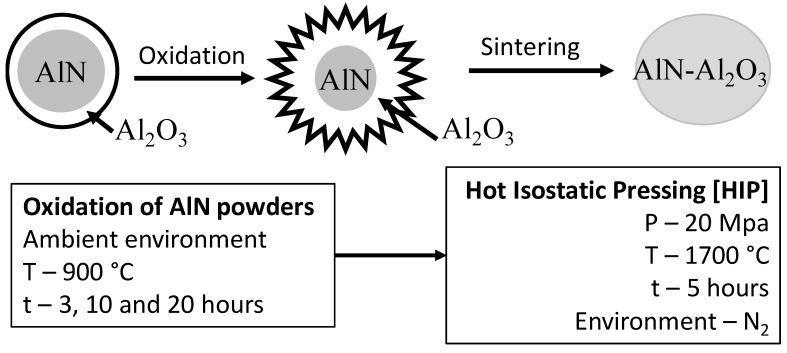
The schematic of oxidation of AlN powder and corresponding sintering process along with the parameters of processing.

**Figure 2:**
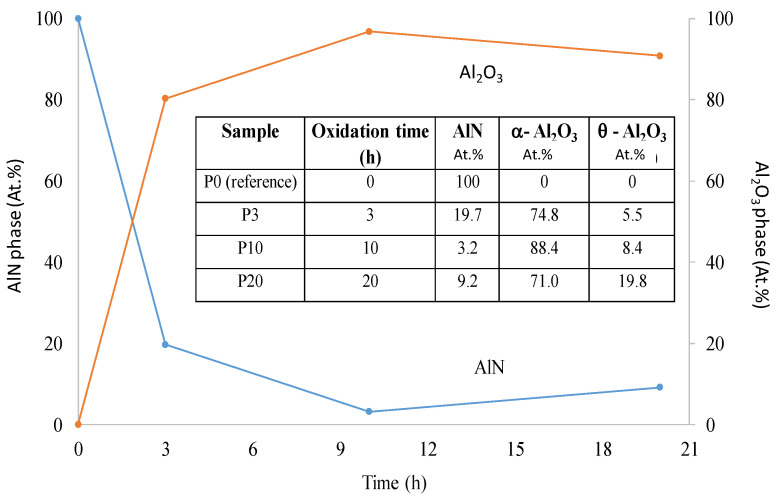
The change and phase transformation observed in the AlN and α-Al_2_O_3_ phases as function of oxidation time as shown in the table in the inset.

The precursors were oxidized in air for different time periods of 3, 10, and 20 h, Figure 1. The percentage of conversion between AlN and Al_2_O_3_ compounds depended on the time of oxidation as presented in Figure 2.

The oxidized powders were then sintered by HIP. The process was carried out in an inert nitrogen atmosphere at a temperature of 1700 °C and pressure of 20 MPa for 5 h. The experiments revealed that the post-sintered samples had reduced porosity and increased apparent density. The measured apparent densities of the sintered AlN-Al_2_O_3_ samples increased over oxidation time (Table 1). Moreover, the oxidation of the precursor AlN powder and the observed phase transformation has certainly helped in the densification of the AlN-Al_2_O_3_ sintered composite.

### 3.2. Morphological Investigation of the Oxidized Powders and Sintered Samples

**Figure 3:**
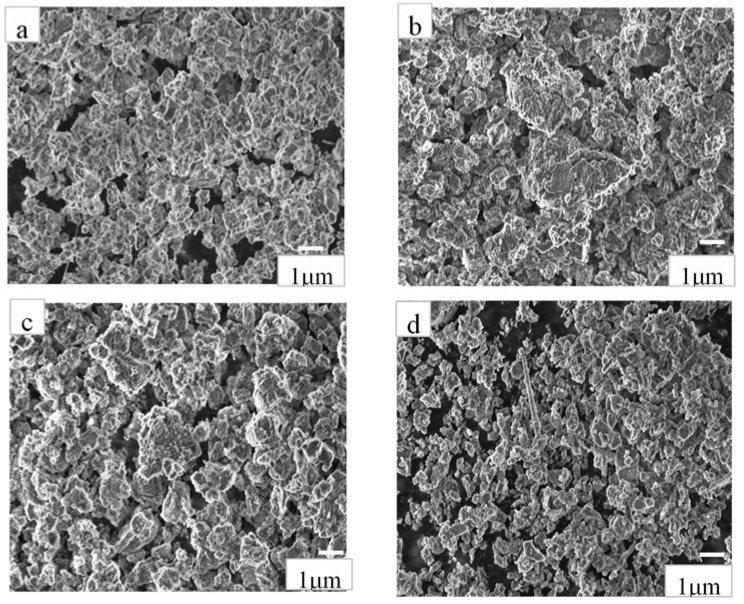
The microstructure of the oxidized powders after respective oxidizing time—(**a**) is the reference AlN powder, (**b**–**d**) are the AlN powder oxidized for 3, 10, and 20 h respectively.

The oxidized powders were structurally characterized using SEM. The powders exhibited increasing oxidation and transition from AlN to Al_2_O_3_ phase. The oxidized AlN powder to AlN-Al_2_O_3_ was observed from SEM micrographs in Figure 3a–d after 0, 3, 10, and 20 h respectively. It is visible that the powders consist of small, irregular shaped particles in the size of several hundred nanometers. The size distribution of particles is homogeneous in the case of reference AlN powder (Figure 3a), however, after 3 and 6 h of oxidation, some agglomerations are also appearing (Figure 3b,c), the particles tended to cohere into larger blocks in micrometer size. The sample after 10 h of oxidation shows very different morphology, in this case the size of the particles varies in wider dimensions. In addition, long, rod-like particles can also be found, indicating the phase transformation to be near complete. The increased oxidation time provides more time for the AlN powder to transform, leading to formation of aluminum oxide. Over the course of oxidized time, the powders exhibit growth of the dual phase AlN-Al_2_O_3_ composite.

**Figure 4:**
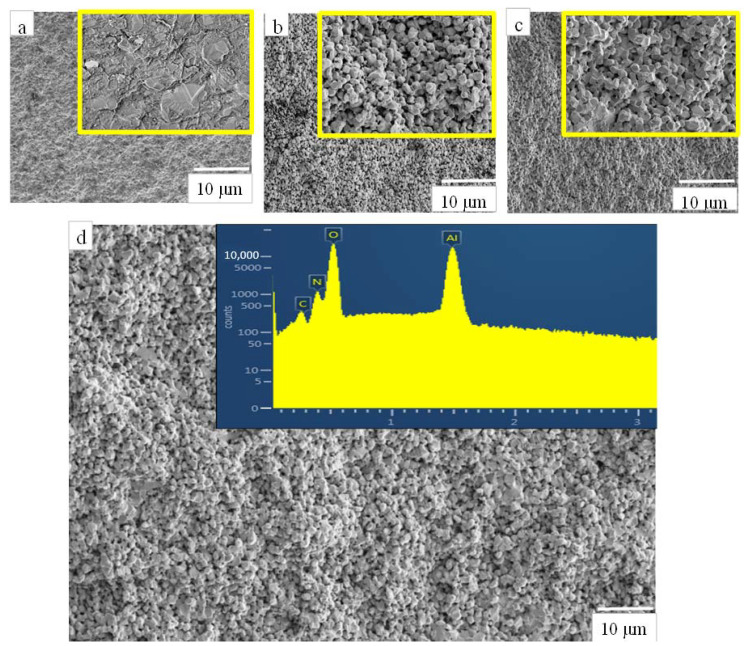
SEM micrographs of Hot Isostatic Pressing sintered AlN-Al_2_O_3_ samples: (**a**–**d**) are after 0, 3, 10, and 20 h of oxidation time along with EDS-spectra of the 20 h sample was also shown in support of the above micrograph indicating the strong presence of Al, O, N peaks. High magnified images (2000×) of the respective images were also provided in the inset.

During the oxidation process of AlN powder, complex dual AlN-Al_2_O_3_ phase was formed and this new phase was then sintered with the given parameters. The sintered samples showed higher density since during the sintering, under the applied pressure and temperature, the samples undergo thermal aging, thus resulting in reduced porosity within the substrate. The HIP sintering method provides homogeneous distribution of pressure across the cross-section of the substrate. The morphology of the sintered samples is demonstrated in Figure 4. As is visible, the size distribution of particles is very homogeneous in all cases. The shape of the particles in the oxidized powders differs significantly compared to reference material after the sintering procedure. The reference material consists of mainly large, agglomerated, and parallelly oriented plate-like particles (Figure 4a), while, for oxidized samples, the shapes of the individual particles are mainly smaller hexagonal blocks. Xue et al. [40] prepared Al_x_O_y_N_z_ from Al_2_O_3_ based ceramics using ball milling and Hot Pressing (HP) sintering at 1800 °C and 20 MPa for 2 h. The parameters used by them were similar to those in our case and the reported densities were comparable. While Xue et al. have reported density of 3.66 g/cc, we have determined the density in our case as 3.12 ± 0.17 g/cc. Similar range of densities for sintered Al_x_O_y_N_z_ ceramics were also reported in Refs [27,31,32,33].

### 3.3. Structural Investigation of the Oxidized Powders and Sintered Samples by XRD

**Figure 5:**
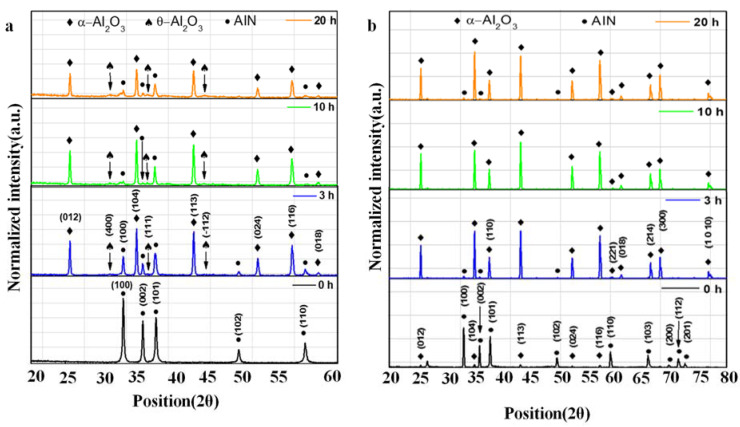
XRD diffractograms of both oxidized powders (**a**) and sintered samples (**b**). Oxidation has resulted in the conversion of pure AlN (at 0 h) to two distinct phases of aluminum oxide namely α-Al_2_O_3_ and θ-Al_2_O_3_ (at 3, 10, and 20 h) in (**a**). The sintered samples on the other hand show presence of only α-Al_2_O_3_ besides AlN, proving that the sintering results in disintegration of θ-Al_2_O_3_ phase.

Figure 5 demonstrates the characteristic X-ray diffraction patterns of powders. It was confirmed that two different phases of aluminum oxide were grown simultaneously, α-Al_2_O_3_ (Rhombohedral-JCP2:00–010-0173) and the intermediary θ-Al_2_O_3_ (Monoclinic-JCP2:01-086-1410) upon oxidation of pure AlN (Hexagonal-JCP2:03-065-1902). Although the second phase of aluminum oxide can be observed only in the powders after 10 and 20 h of oxidation time. Since aluminum and aluminum nitride readily react with oxygen, the probability of transformation of AlN to Al_2_O_3_ is high. Some research works revealed that the intermediary phase has about 35.7 at% AlN and 64.3 at% Al_2_O_3_ at equilibrium [5]. Though XRD analysis shows formation of two phases of aluminum oxide, the dominant phase is α-Al_2_O_3_. In our XRD diffractograms of the oxidized powders, the signal pertaining to θ-Al_2_O_3_ at 30°, 36°, and 44° of 2θ position can only be seen in the oxidized powders at 10 and 20 h’ oxidation time. The intermediary aluminum oxide phase, θ-Al_2_O_3_ was reported in other studies [41,42,43,44], which is in agreement with our results. The most commonly agreed fact is that AlN is mostly oxidation-resistant at temperatures below 850 °C. The initial oxidation of AlN exhibits a linear behavior and proceeds by formation of discontinuous and thin protective oxide film at the surface. This thin film is mostly porous and hence acts as a further oxidation site [41]. As the temperature increase above 950 °C, the AlN surface becomes more susceptible to oxidation. Above 1000 °C, the oxidation of the surface occurs abruptly and a thick film begins to form at the surface. Above 1050 °C, the oxidation follows a parabolic trend [42,43,44]. In our experiments, at 900 °C, α-Al_2_O_3_ was formed exhibiting a coarse structure on the surface (Figure 3). Generally, α-Al_2_O_3_ is a thermodynamically stable structure of the aluminum oxide phases with a rhombohedral crystal structure. Other structures of alumina include monoclinic θ-Al_2_O_3_ and tetragonal δ-Al_2_O_3_ which are usually transition phases of alumina as investigated by Duchesne and Hipps [45] and Gu and Edgar [46]. The presence of θ-Al_2_O_3_ is proof of the formation of transition alumina and hence the intermediary oxynitride generation in the powders.

On the other hand, the XRD analysis of sintered samples showed the presence of AlN and α-Al_2_O_3_ phases, Figure 5b. We can confirm that the θ-Al_2_O_3_ phase which was significantly present in the oxidized AlN powders was absent in the sintered samples. The reason for this absence was reported by Gu and Edgar [46]. They concluded that the transition oxide states of alumina such as θ-Al_2_O_3_ are metastable and disintegrate at temperatures above 1100 °C. The peaks of signals can be seen varying considerably with the XRD analysis as function of time of oxidation in the samples. At 0 h, we see only the presence of AlN while the 10 and 20-h samples have shown almost presence only of Al_2_O_3_ indicating complete transformation.

### 3.4. Mechanical Testing of the HIP Sintered Composites

**Figure 6:**
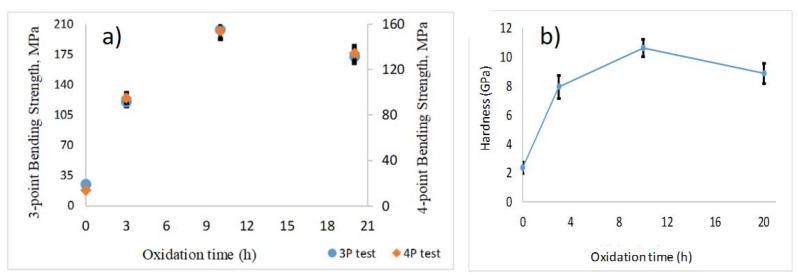
The measured 3-point and 4-point bending strength (**a**) and micro-hardness (**b**) of the sintered AlN-Al_2_O_3_ given as function of oxidizing time of the samples.

The sintered composites were tested for their mechanical properties such as micro-hardness test and bending tests to quantify their mechanical strength and hardness. Both three-point and four-point bending tests conducted on the sintered oxidized composites showed an increase in the bending strength of the samples compared to the non-oxidized samples. Similar trend in results was observed in hardness test. Both sets of values increased as function of oxidation time initially provided for the AlN powders and the plots can be seen in Figure 6.

The bending strength of the sintered samples according to the 3-point bending test was ~25 MPa (S0—for AlN, non-oxidized sample) and 204 MPa (S10—sintered after 10-h oxidation). The values for 4-point bending tests were ~14 MPa (S0—for AlN, non-oxidized sample) and 154 MPa (S10—sintered after 10-h oxidation, see Figure 6a). The interesting observation in these test results is that the values of bending test in both the cases (3-point and 4-point) were found to be highest for S10 samples compared to S20 samples. The hardness tests on the sintered samples for micro-hardness yielded the values in the range of 8 and 11 GPa, Figure 6b. These mechanical characteristic results can be attributed to the unique microstructure of AlN-Al_2_O_3_ composite after 10 and 20 h sintering process. As it was clearly visible in Figure 4, the shape of the particles changed from plate-like to small, compact hexagonal cubes owing to the sintering and the particle sizes also decreased significantly. The distribution of particle sizes was very homogeneous, they formed a compact structure with almost no pores present which gave the composite higher mechanical strength. According to the mechanical tests, this compaction was the highest in the case of sample oxidized for 10 h. In an earlier work, the micro hardness of AlN-Al_2_O_3_ system was found in the range of 12–20 GPa as reported by Spivak et al. [47]. Hardness values of AlN vary between 9–10 GPa [42] whereas for Al_2_O_3_ it ranges between 17 and 23 GPa [48]. The reported values for the AlN-Al_2_O_3_ sintered ceramics in our case were in the range between the two terminal values. However, a slight decrease in the values of hardness and bending strength were observed in S20 sample. This decrease in the values correspond to the slight increase in the composition of AlN in the S20 samples as compared to the S10 samples (Figure 2).

The generally observed trend of improved mechanical properties of the sintered AlN-Al_2_O_3_ samples were attributed to the refinement in the microstructure as reported by Swab et al. and others [49,50,51]. The dispersion of the aluminum oxide in the matrix has been shown to improve the microhardness already in the metal matrix composites [52,53,54]. A similar effect has been observed in our case as the transformation of AlN to Al_2_O_3_ has imparted strength to the sintered composite. HIP sintering helps in the dispersion of the Al_2_O_3_ phase across the surface of the AlN substrate. The grain boundaries are imparted by dispersion strengthening mechanism and in turn improves the microhardness and bending strength.

### 3.5. Tribological Investigations on AlN-Al_2_O_3_ Samples

#### 3.5.1. Experimental Investigations

**Figure 7:**
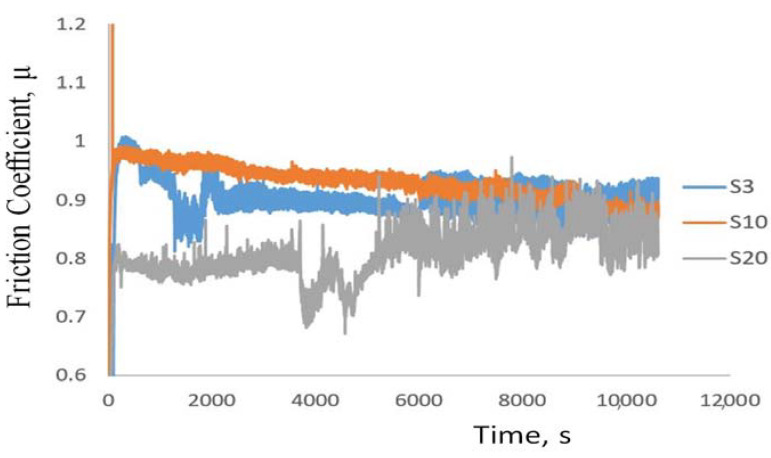
Friction coefficient of various sintered samples as function of time.

**Figure 8:**
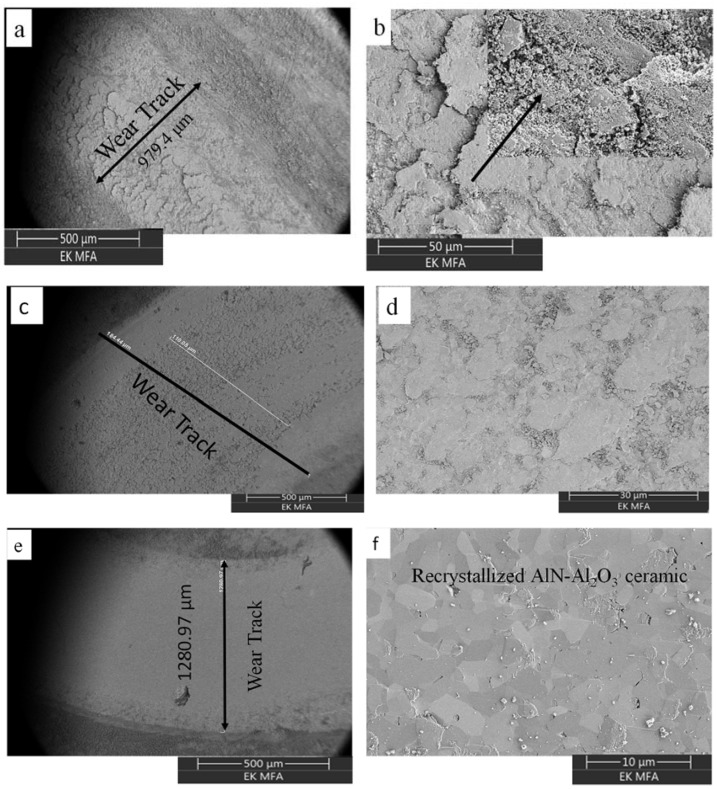
SEM micrographs of the wear track after the tribology tests. The (**a**,**b**) after 3 h oxidation time—S3, (**c**,**d**) after 10 h oxidation time—S10, (**e**,**f**) after 20 h oxidation time—S20 and respectively. We also see Al_2_O_3_ oxide clusters at the surface of the wear track in the inset of (**b**) and the recrystallized AlN-Al_2_O_3_ dual composite at the surface of (**f**).

Tribological measurements were also performed on the sintered AlN-Al_2_O_3_ samples under the conditions as mentioned in Section 2. While the tests performed on the sintered oxidized samples were successful, the test performed on sintered AlN reference sample broke during experimentation and hence could not be quantified. The real time coefficient of friction between the disc and the ball was measured during the experiment. The friction coefficient measured during the experiment was plotted versus the time and was observed to be slightly higher than expected (Figure 7). The friction coefficients of the oxidized and sintered samples were found to be in the range of 0.9 ± 0.2 compared to the friction coefficient of AlN which is about 0.3 ± 0.1 [55]. On the other hand, the coefficient of friction of Al_2_O_3_ on average is around 0.6–0.8 [56]. The higher values could be because of the clusters of aluminum oxide formed at the surface of the sintered substrates along with AlN-Al_2_O_3_ clusters, Figure 8.

The wear track width was measured using optical microscopy, the wear parameters like wear rate and wear factor (Equations (2) and (3)) were calculated and tabulated, Table 2.

The constant parameters required for calculations are radius of the track R—3.5 mm, radius of the ball r—2.5mm, load used—5N and sliding distance—2,000,000 m.

#### 3.5.2. Morphological Study on Samples Subjected to Tribology Measurements

The surface of the samples was analyzed by scanning electron microscopy (SEM) to observe the compositional changes occurring at the surface (Figure 8). We observed that the morphology of the wear track depended on the time of oxidation. In Figure 8a–d after 3, 10, and 20 h of oxidation time respectively, the surface is coarse which indicates the lower percentage conversion of the AlN to Al_2_O_3_. In Figure 8e,f the morphology of the surface has a more fine and smooth texture, indicating the near complete conversion of AlN to Al_2_O_3_ phase and equilibrium dispersion within the substrate. The AlN-Al_2_O_3_ phases can be seen at the surface of S20 sample (Figure 8f) which can explain the improved hardness and bending strength exhibited by the sample. Furthermore, the extensive tribofilm formation, brittleness, and surface cracks observed during the experimentation are due to the presence of clusters of Al_2_O_3_ at the surface as seen on the inset of Figure 8b. The extensive tribofilm formation provided a stable friction behavior (S3), whereas the lower amount of tribofilm formation (S10) or no tribofilm formation on smooth surface (S20) resulted in unstable frictional behavior (Figure 7).

**Figure 9:**
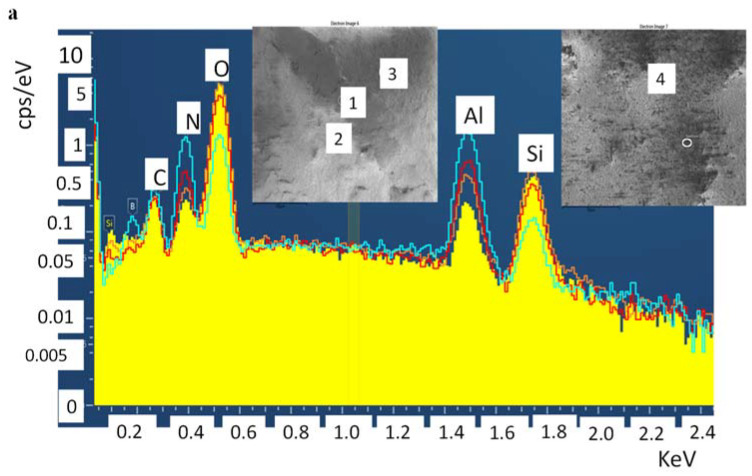

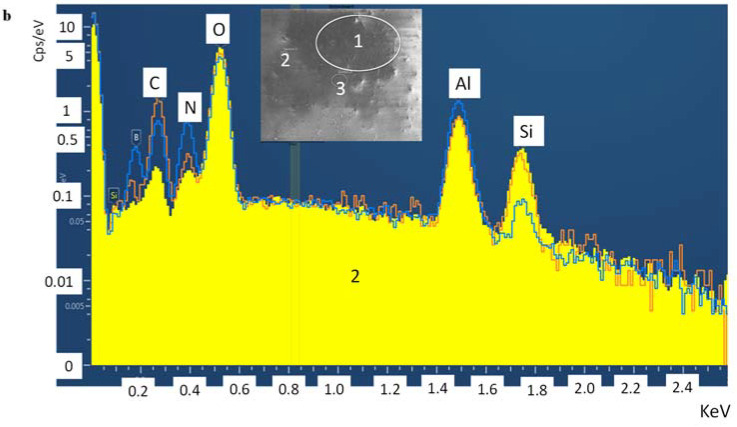
SEM-EDS spectra of various areas in and around the tribological tracks of S3 (**a**) and S20 (**b**). The presence of Al, O, N can be detected in both the samples along with residuals like Si (from abrasion of Si_3_N_4_ counterpart) and C (from carbon tape in SEM).

The compositional analysis of the area within the wear track and just outside the wear track was collected using SEM-EDS. Presence of dual AlN-Al_2_O_3_ was identified in all cases, Figure 9. This dual phase presence confirms the partial conversion under insufficient/low oxidation time (3 h). The tribofilm formation, coarsening, and cracking observed at the surfaces of samples after tribology test can be attributed to partial aluminum oxide clustering as shown in Figure 8b (inset). Thus, the samples S3 have exhibited comparatively poor tribological properties and higher volume of wear (Table 2). On the contrary, samples S10 and S20, sintered after 10-h and 20-h oxidation of the AlN powder, showed partial or total smooth morphology. The near complete conversion of the AlN to Al_2_O_3_ phase has strengthened the substrate and can be understood as part reason why we observe no cracks at the surface post experimentation. Furthermore, from SEM-micrographs we see rearranged or redistributed Al_2_O_3_ grains as seen in Figure 8f. The rearrangement has imparted the necessary hardness to the surface leading to the lower friction coefficients observed (S20, Figure 7).

The SEM-EDS spectra in Figure 9 show the presence of both Al, N, O elements or AlN and Al_2_O_3_ phases in the substrate. However, Figure 9a belongs to S3 and Figure 9b belongs to S20. We can observe the difference in the intensity of the O and N in the Figure 9a,b which show the difference in conversion. Here it is important to note that the residual signals Si comes from the abrading action of the Si_3_N_4_ ball on the substrate surfaces during tribology test.

## 4. Conclusions

- The preparation of composite AlN-Al_2_O_3_ ceramics was carried out by oxidation of pure AlN powders and subsequent hot isostatic pressing.
- The oxidized AlN powders have developed two distinct phases of aluminum oxide (α-Al_2_O_3_ and θ-Al_2_O_3_). The transformation depended on the time of oxidation with lower percentage of conversion obtained after 3 h and near complete transformation of AlN to Al_2_O_3_ at 20-h oxidation.
- The sintered samples showed the presence of only α-Al_2_O_3_ besides AlN proving that the sintering results in disintegration of θ-Al_2_O_3_ phase.
- Upon sintering, the substrates showed marked improvement in the density with a reduction in the porosity. The experiments also proved that densification in sintered samples can be achieved by HIP sintering particularly at lower temperatures (<1800 °C). The measured density values were in agreement with values reported in literature.
- Mechanical tests showed considerable improvement in the bending strength and microhardness of the sintered substrates. However, superior properties in both the cases were observed in samples S10 followed by S20.
- According to the tribology tests, the samples showed considerably good wear property. Furthermore, the extensive tribofilm formation, brittleness, and surface cracks observed during the experimentation are due to the presence of clusters of Al_2_O_3_ at the AlN surface. The extensive tribofilm formation provided a stable friction behavior (S3), whereas the lower amount of tribofilm (S10) or no tribofilm formation on smooth surface (S20) resulted in unstable frictional behavior.

## Figures and Tables

**Table 1:** Apparent density of the sintered samples shown as function of oxidation time.

Sample	Oxidation Time of Sample (h)	Apparent Density (g/cm^3^)
S0	0	2.57
S3	3	2.87
S10	10	3.37
S20	20	3.10

**Table 2:** Wear track width measured from optical microscopy and volume wear and wear factor calculated using Equations (2) and (3).

Sample	Wear Track Width, d (mm)	Volume Wear, V (mm^3^)	Wear Factor, K (mm^2^/N)
S3	1.434	2252.95	0.000225
S10	1.575	2482.5	0.000248
S20	0.954	1486.1	0.000149
